# The impact of iron deposition on the fear circuit of the brain in patients with Parkinson’s disease and anxiety

**DOI:** 10.3389/fnagi.2023.1116516

**Published:** 2023-02-09

**Authors:** Kaidong Chen, Li Zhang, Haixia Mao, Kefei Chen, Yachen Shi, Xiangpan Meng, Feng Wang, Xiaoyun Hu, Xiangming Fang

**Affiliations:** ^1^Department of Radiology, The Affiliated Wuxi People’s Hospital of Nanjing Medical University, Wuxi, China; ^2^Department of Neurology, The Affiliated Wuxi People’s Hospital of Nanjing Medical University, Wuxi, China

**Keywords:** quantitative susceptibility mapping, anxiety, Parkinson’s disease, fear circuit, brain iron deposition

## Abstract

**Objective:**

Anxiety is one of the most common psychiatric symptoms of Parkinson’s disease (PD), and brain iron deposition is considered to be one of the pathological mechanisms of PD. The objective of this study was to explore alterations in brain iron deposition in PD patients with anxiety compared to PD patients without anxiety, especially in the fear circuit.

**Methods:**

Sixteen PD patients with anxiety, 23 PD patients without anxiety, and 26 healthy elderly controls were enrolled prospectively. All subjects underwent neuropsychological assessments and brain magnetic resonance imaging (MRI) examinations. Voxel-based morphometry (VBM) was used to study morphological brain differences between the groups. Quantitative susceptibility mapping (QSM), an MRI technique capable of quantifying susceptibility changes in brain tissue, was used to compare susceptibility changes in the whole brain among the three groups. The correlations between brain susceptibility changes and anxiety scores quantified using the Hamilton Anxiety Rating Scale (HAMA) were compared and analyzed.

**Results:**

PD patients with anxiety had a longer duration of PD and higher HAMA scores than PD patients without anxiety. No morphological brain differences were observed between the groups. In contrast, voxel-based and ROI-based QSM analyses showed that PD patients with anxiety had significantly increased QSM values in the medial prefrontal cortex, anterior cingulate cortex, hippocampus, precuneus, and angular cortex. Furthermore, the QSM values of some of these brain regions were positively correlated with the HAMA scores (medial prefrontal cortex: *r* = 0.255, *p* = 0.04; anterior cingulate cortex: *r* = 0.381, *p* < 0.01; hippocampus: *r* = 0.496, *p* < 0.01).

**Conclusion:**

Our findings support the idea that anxiety in PD is associated with iron burden in the brain fear circuit, providing a possible new approach to explaining the potential neural mechanism of anxiety in PD.

## 1. Introduction

Parkinson’s disease (PD) is a common neurodegenerative disease characterized by motor disorders such as bradykinesia, resting tremor, muscular rigidity, and postural instability (Postuma et al., 2015). It has recently been found that PD is also closely related to several psychiatric symptoms, which have a remarkable influence on health-related quality of life (Carod-Artal et al., 2008; Martínez-Martín and Damián, 2010; Gallagher and Schrag, 2012). Anxiety occurs more frequently in PD patients than in patients suffering from other chronic neurodegenerative diseases or in elderly controls with similar disabilities (Pontoon et al., 2009; Martínez-Martín and Damián, 2010). Although PD patients experience anxiety (Carod-Artal et al., 2008), the potential mechanism of anxiety in PD remains a matter of debate.

Anxiety and fear are thought to share a similar neural circuit, called the ‘fear circuit’, where fear as a feeling is experienced in response to existing danger or threat, while anxiety is experienced in response to prospective or imagined future danger or threat (Penninx et al., 2021). The fear circuit is composed of the amygdala, medial prefrontal cortex (mPFC), anterior cingulate cortex (ACC), hippocampus, insula, and striatum (Dresler et al., 2013). As the input and output nuclei of the circuit, the amygdala plays a central role in detecting external threats, transmitting information to other nodes of the fear circuit, and eventually exporting negative emotions such as anxiety and fear (Sun et al., 2020). Other areas of the fear circuit regulate the amygdala through ‘top-down’ cognitive processes, including assessing emotional threats, distracting attention from threats, and learning or extinction fear conditioning memory (Etkin et al., 2011; Mah et al., 2016). In previous studies, dysfunction of the fear circuit has been used to explain the neural mechanism of anxiety in PD (Carey et al., 2021). For example, to date, functional magnetic resonance imaging (fMRI) (Carey et al., 2020; Criaud et al., 2021) and voxel-based morphometry (VBM) (Vriend et al., 2016) studies have reported anatomical or functional alterations in key brain areas of the fear circuit in patients with PD and anxiety. However, the underlying mechanisms of these brain abnormalities remain unclear.

An increasing number of studies have found that aberrant iron metabolism is involved in PD pathogenesis. Excessive iron deposition in the brain, decreased serum iron levels, and increased transferrin levels in the circulation have been observed in patients with PD (Dexter et al., 1991; Yoshihara et al., 2016; Xu et al., 2018; Mochizuki et al., 2020). High-level transferrin transfers iron to the brain, especially the substantia nigra (SN) (Dexter et al., 1991; Costa-Mallen et al., 2017), by translocating iron-loaded transferrin and its receptor to the intracellular compartment (Kaur and Andersen, 2004). Oxidative stress and aggregation of α-synuclein (Ostrerova-Golts et al., 2000) caused by excessive iron deposition in the SN are considered to be involved in the pathogenesis of PD (Ayton and Lei, 2014).

Abnormal iron metabolism has also been shown to be closely associated with anxiety in animal experiments. Increased anxiety-like behaviors have been observed in iron-deficient rats (Beard et al., 2002; Li et al., 2011). After iron treatment, excessive iron deposition was observed in rat brain regions, such as the basal ganglia, frontal cortex, hippocampus, and cerebellum, and was accompanied by increased anxiety behaviors (Maaroufi et al., 2009). In PD patients, low serum iron and high serum transferrin levels are significantly associated with the severity of anxiety (Xu et al., 2018).

Therefore, we assumed that PD patients with anxiety have more severe iron deposition in the brain regions of the fear circuit than PD patients without anxiety or healthy individuals. Furthermore, oxidative stress caused by iron deposition in these brain regions leads to cellular and tissue damage (Nandar et al., 2013), which may be one of the fundamental causes of anxiety in PD. Perhaps because of the invasiveness of previous detection methods, few studies have focused on iron deposition in these brain regions in PD patients with anxiety. However, with the development of MRI technology, noninvasive and accurate quantitative detection of iron deposition in the brain has become possible.

Quantitative susceptibility mapping (QSM) is an emerging MRI technique capable of quantifying susceptibility changes in the brain tissue (Port and Pomper, 2000). Although other metals, such as aluminum and copper, may also result in increased susceptibility measurements, it can still be considered that iron is the most important factor affecting susceptibility differences because the effects of other metals are too mild compared with those of iron in the brain (Becker et al., 2005; Popescu et al., 2009). At present, QSM has been widely used in various aspects of PD research as imaging biomarkers and plays a crucial role in the diagnosis (Xiao et al., 2019), staging (Li et al., 2021), and assessment of motor (Chen et al., 2020) and non-motor (Uchida et al., 2019; Thomas et al., 2020) symptoms in PD. However, no studies to date have used QSM to explore the association between brain iron deposition in the fear circuit and anxiety in PD patients. Furthermore, the distribution of brain iron in patients with PD and anxiety remains unclear.

In this study, we hypothesized that there would be more iron accumulation in the brain regions of the fear circuit in PD patients with anxiety. Here, voxel-based QSM analysis was applied to compare the susceptibility of the whole brain among the three groups, i.e., PD patients with anxiety (PD-A), PD patients without anxiety (PD-NA), and healthy controls (HC). Then, based on the region of interest (ROI)-based analysis, regional QSM values related to the fear circuit were obtained and compared among the three groups, and the correlation between the QSM values of these brain regions and anxiety scores was determined. In this study, we aimed to comprehensively investigate differences in regional magnetic susceptibility among the three groups and explore the role of iron in the potential neural mechanisms of anxiety in PD.

## 2. Materials and methods

### 2.1. Subjects

Forty-one patients with PD were prospectively and consecutively enrolled in this study from the Department of Neurology, Affiliated Wuxi People’s Hospital of Nanjing Medical University (Wuxi, China) between August 2021 and August 2022. Twenty-six age-and sex-matched healthy individuals without anxiety were enrolled as controls. This study was approved by the Ethics Committee of WuXi People’s Hospital and was conducted in accordance with the Declaration of Helsinki. Written informed consent was obtained from all the participants. To reduce the impact of drugs in this study, drugs used to treat PD were banned at least 12 h before the MRI examination.

The inclusion criteria for PD patients in this study were (1) diagnosis of PD based on the UK PD Brain Bank criteria (Postuma et al., 2015), (2) age > 40 years, (3) right-handed, (4) ability to complete MRI examination and neuropsychological assessment, and (5) voluntary participation in this study.

The exclusion criteria for patients with PD in this study were (1) any history of head injury, cerebrovascular diseases, or severe neurological diseases; (2) cognitive impairment; (3) abnormal findings on brain imaging; and (4) poor imaging quality.

### 2.2. Clinical assessments

Participant demographics such as age, sex, education, disease duration, and levodopa equivalent daily dose (LEDD) were collected from patients with PD after completing MRI scanning. Two neurologists with years of clinical experience performed detailed motor and neuropsychological assessments of the participants: (1) Movement Disorder Society Unified PD Rating Scale motor part 3 (MDS-UPDRS-III) to evaluate the severity of PD motor symptoms; (2) Hoehn & Yahr scales (H&Y) to evaluate the PD disease stage; (3) Hamilton Anxiety Rating Scale (HAMA) and 17-item Hamilton Depression Rating Scale (HAMD) to evaluate the mental state of PD patients; (4) Mini-Mental State Examination (MMSE) to evaluate cognitive function in PD patients; (5) Frontal Assessment Battery (FAB) to evaluate the frontal executive function of PD patients, and (6) Freezing of Gait Questionnaire (FOGQ) to assess the correlation between freezing of gait and anxiety. PD patients were divided into the PD-A group if (1) meeting the diagnostic criteria of anxiety as defined by the DSM-IV (Diagnostic and Statistical Manual of Mental Disorders, fourth edition) criteria (American Psychiatric Association, 1995) and (2) HAMA scores ≥12 (Leentjens et al., 2011; Wang et al., 2017). For the healthy controls, we also collected MRI data, demographic characteristics, and partial scales such as HAMA, HAMD, and MMSE through the same process.

### 2.3. Image acquisition

MRI scans were obtained in the morning from each participant on a 3.0 T Siemens Prisma whole-body MRI system (Magnetom 3T Siemens, Prisma, Germany) using a 20-channel phased-array head coil. When adjusting the lying position, foam pads and earplugs were used to minimize head motion and scanner noise.

To assess susceptibility information in the brain, the magnitude and phase images were obtained by running a three-dimensional (3D) fast low-angle shot sequence with four echoes using the following parameters: repetition time (TR) = 35 ms, echo time (TE): TE1 = 7.5 ms; TE2 = 14.42 ms; TE3 = 21.34 ms; TE4 = 28.26 ms, flip angle (FA) = 20°, slice thickness = 1 mm, field of view (FOV) = 220 × 220 × 128 mm^2^, matrix size = 220 × 220 mm^2^, voxel resolution = 1 × 1 × 1 mm^3^, and acquisition time (TA) = 7 min 21 s.

To acquire anatomical information about the brain, a volumetric 3D-T1 magnetization prepared rapid acquisition gradient echo (MP-RAGE) sequence was run using the following parameters: TR = 2,300 ms, TE = 2.98 ms, inversion time (TI) = 900 ms, FA = 9°, slice thickness = 1 mm, FOV = 256 × 256 × 192 mm^3^, matrix size = 256 × 256 mm^2^, voxel resolution = 1 × 1 × 1 mm^3^, and TA = 5 min 30 s.

To rule out any brain abnormalities, T2-weighted, fluid-attenuated inversion recovery (FLAIR), diffusion-weighted (DWI), and magnetic resonance angiography (MRA) images were obtained.

### 2.4. Voxel-based morphometry analysis

Computational Anatomy Toolbox (CAT) 12 software (Kurth et al., 2015) was used to conduct the VBM analysis to explore the morphological differences among the three groups on the MATLAB R2016b platform (The MathWorks Inc., Natick, MA, USA). First, the total intracranial volume (TIV) of each participant was calculated from the 3D-T1WI MP-RAGE image. Next, 3D-T1WI MP-RAGE images were segmented into gray matter, white matter, and cerebrospinal fluid. The obtained gray matter images were then spatially normalized and transformed into Montreal Neurological Institute (MNI) space. Subsequently, an 8 × 8 × 8 mm^3^ full width at half maximum Gaussian kernel was used for spatial smoothing of the normalized gray matter images. In addition, the average gray matter image was extracted as a binary gray matter mask.

### 2.5. QSM reconstruction

The quantitative susceptibility maps were calculated using the STISuite toolbox^1^ on the MATLAB R2016b platform, which consists of phase unwrapping, binary brain mask manufacture, background field removal, and dipole inversion. A total field map was calculated by extracting and unwrapping the phase images using the Laplacian-based phase-unwrapping method (Bagher-Ebadian et al., 2008). The FSL^2^ was used to extract the binary brain mask from the first echo magnitude image of each subject. The total field maps and magnitude images for each echo were averaged. To remove the uneven background field, variable-kernel sophisticated harmonic artifact reduction for phase data (V-SHARP) was applied (Wu et al., 2012). The quantitative susceptibility maps were then reconstructed by streaking artifact reduction (STAR) for QSM (Wei et al., 2015).

### 2.6. Voxel-based QSM analysis

Spatial normalization of the quantitative susceptibility maps was performed using Advanced Normalization Tools (ANTs).^3^ First, the first echo magnitude image was co-registered with the 3D-T1WI MP-RAGE image. The quantitative susceptibility map was then transformed according to the co-registration between the first echo magnitude image and the 3D-T1WI MP-RAGE image. Finally, we used the normalized parameters of the 3D-T1WI MP-RAGE image to spatially normalize and transform the co-registered quantitative susceptibility map of each subject into MNI space. Spatial smoothing was performed for the normalized quantitative susceptibility maps using an 8 × 8 × 8 mm^3^ full-width-at-half-maximum Gaussian kernel.

### 2.7. ROI-based QSM analysis

To avoid possible errors in the spatial normalization of the quantitative susceptibility maps and spatial uncertainty in the resulting clusters after smoothing (Uchida et al., 2019), ROI-based QSM analysis aimed to more accurately compare the QSM values of the brain regions related to the fear circuit or known to have rich iron content among the PD-A, PD-NA, and HC groups. ROIs related to the fear circuit were set to correspond to the amygdala, ACC, mPFC, hippocampus, striatum, and insula. Assuming that oxidative stress due to iron accumulation is the mechanism of anxiety, these brain regions may be related to increased QSM values. Iron deposition-based ROIs were set corresponding to the SN, globus pallidus (GP), and red nucleus (RN). All ROIs were obtained from an Anatomical Automatic Labeling (AAL) template (Rolls et al., 2020). In addition, in order to confirm the robustness of the results, the ROIs related to the fear circuit obtained from another brain atlas called Brainnetome Atlas^4^ were used to validate our results. The mean QSM values of the nine defined ROIs were extracted from the quantitative susceptibility maps without smoothing on the MATLAB R2016b platform.

### 2.8. Statistical analysis

Statistical analyses were performed using For Statistical Product Service Solutions (SPSS) version 26.0. All continuous data are expressed as mean ± standard deviation, and discontinuous data are expressed as proportions. After evaluating homoscedasticity and normality, a one-way analysis of variance (ANOVA), two-sample *t*-test, Chi-square test, and Kruskal–Wallis test were performed to compare the demographic and clinical characteristics of the PD-A, PD-NA, and HC groups. Differences were considered statistically significant at a Bonferroni-corrected *p* < 0.05.

Using Statistical Parametric Mapping (SPM) 12 software, a voxel-wise analysis of covariance (ANCOVA) test within the gray matter mask obtained from the VBM analysis was applied, with age, sex, years of education, PD disease duration, HAMD scores, and TIV as covariates, to compare the voxel-based QSM values among the PD-A, PD-NA, and HC groups, followed by post-hoc two-sample *t*-tests in the brain regions with significant intergroup differences. A voxel-wise *p* < 0.001 and a cluster-wise family wise error (FWE)-corrected p < 0.05 was set as the significance level. Then, we conducted the statistical analysis of the VBM analysis in the same way as the voxel-wise QSM analysis. By using SPSS 26.0 software, the mean QSM values of each ROI among the three groups were compared using an ANCOVA with adjustment for age, sex, years of education, HAMD scores, and PD disease duration after evaluating normality. Post-hoc tests were performed with Bonferroni correction for multiple comparisons at a significance level of *p* < 0.05.

**Figure 1:**
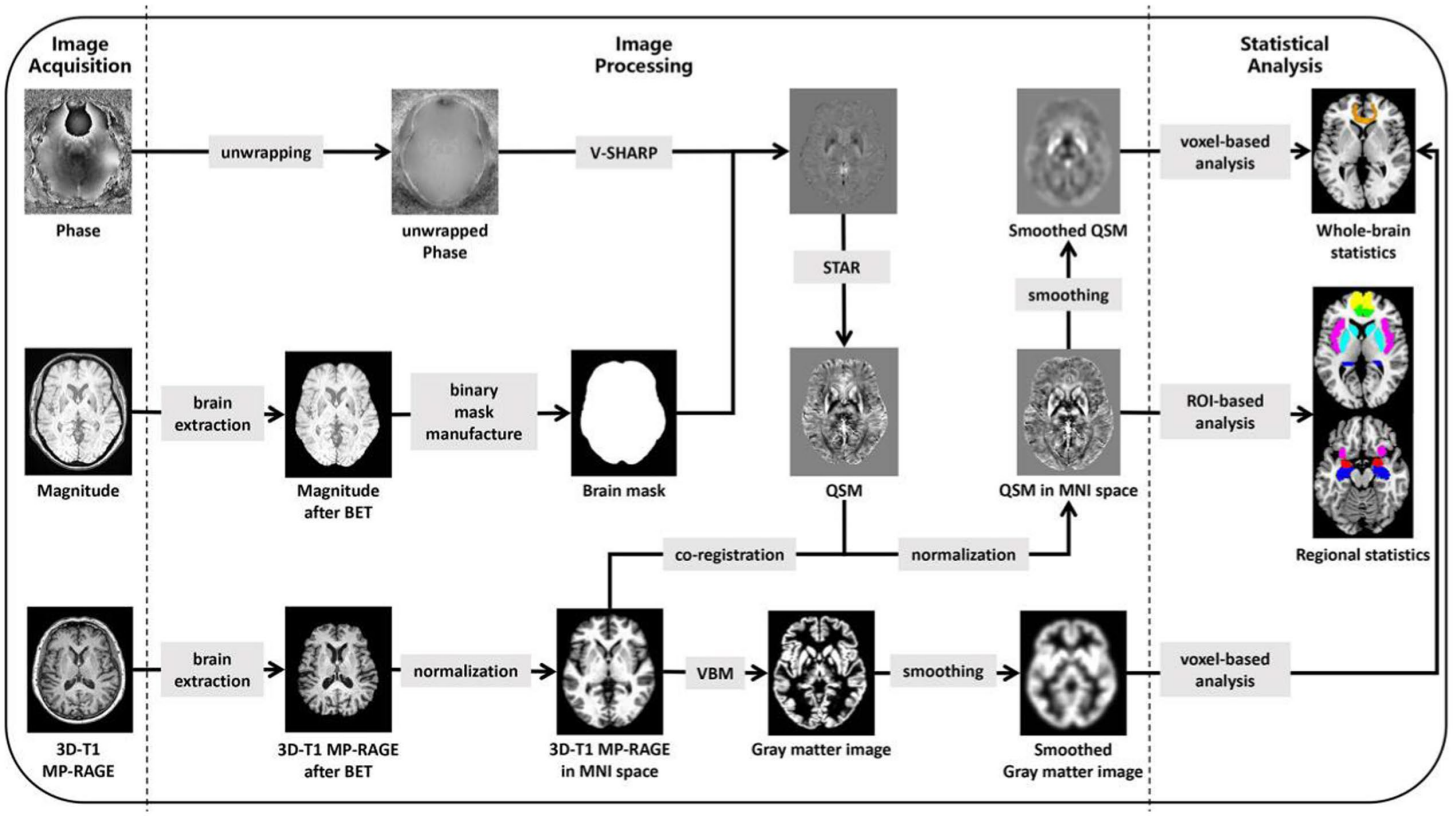
Summary steps of the processing for images and statistical analysis. 3D, three-dimensional; MP-RAGE, magnetization prepared rapid acquisition gradient echo; BET, brain extraction tool; V-SHARP, variable-kernel sophisticated harmonic artefact reduction for phase data; STAR, streaking artifact reduction; QSM, Quantitative Susceptibility Mapping; MNI, Montreal Neurological Institute; VBM, voxel-based morphometry; ROI, based on the region of interest.

Finally, a correlation analysis between voxel-wise QSM values and HAMA scores was performed for all subjects using SPM12 after adjusting for age, sex, years of education, PD disease duration, HAMD scores, and TIV. The significance level was set at voxel-wise p < 0.001 and cluster-wise FWE-corrected p < 0.05. The mean QSM values of the statistically significant clusters, defined as additional ROIs, were also extracted. Then, after the evaluation of data normality, Pearson’s correlation analysis was performed to investigate the relationship between the mean QSM values of each ROI and HAMA scores in all subjects using SPSS 26.0. Additionally, the correlations between HAMA scores and QSM values in the single PD-A group were analyzed by the same process. For a summary of imaging processing steps and statistical analysis, see Figure 1.

## 3. Results

### 3.1. Population

Two participants were excluded due to poor imaging quality. This study included 39 patients with PD and 26 healthy controls. The subjects were divided into PD-A (*n* = 16), PD-NA (*n* = 23), and HC (*n* = 26) groups. All subjects in the PD-A group developed anxiety symptoms after PD.

### 3.2. Demographic and clinical characteristics

No significant differences were found in age, sex, education, MMSE, FAB, or TIV among the three study groups (*p* > 0.05, Bonferroni correction). Compared with the PD-NA group, the PD-A group showed no significant difference in terms of the LEDD, H&Y stage, FOGQ, and UPDRS-III scores (*p* > 0.05). However, the PD-A group had a longer PD duration and a higher H&Y state than the PD-NA group (*p* < 0.05). Additionally, the HAMA and HAMD scores of subjects in the PD-A group were statistically higher than those in the PD-NA and HC groups (*p* < 0.01) but showed no statistical differences between the PD-NA and HC groups (*p* > 0.05). All details are displayed in Table 1.

**Table 1:** Comparison of demographics and clinical characteristics among the PD-A, PD-NA and HC groups.

	PD-A (*n* = 16)	PD-NA (*n* = 23)	HC (*n* = 26)	*p* value
Sex (M/F)	6/10	15/8	14/12	0.233^a^
Age (years)	66.94 ± 7.22	63.17 ± 8.54	62.15 ± 8.94	0.205^b^
Education (years)	10.53 ± 2.17	10.50 ± 3.06	10.54 ± 4.14	0.898^c^
LEDD (mg)	438.28 ± 51.54	328.26 ± 121.67	NA	0.068^d^
Disease duration (years)	5.91 ± 3.52	3.17 ± 2.22	NA	0.011^d,^*
H&Y	2.31 ± 0.75	1.91 ± 0.73	NA	0.106^d^
UPDRS-III	26.00 ± 12.61	22.04 ± 12.03	NA	0.328^d^
FOGQ	5.88 ± 7.65	2.21 ± 4.34	NA	0.098^d^
HAMA	19.06 ± 4.96	6.04 ± 2.57	5.00 ± 2.06	<0.001^b,^*
HAMD	12.31 ± 5.29	4.61 ± 3.07	4.23 ± 2.12	<0.001^b,^*
MMSE	28.69 ± 1.38	28.83 ± 1.11	29.12 ± 0.86	0.634^c^
FAB	17.13 ± 1.26	17.35 ± 0.71	17.65 ± 0.56	0.243^c^
TIV (ml)	1436.36 ± 204.48	1493.79 ± 171.91	1427.32 ± 141.23	0.361^c^

PD-A, Parkinson’s disease with anxiety; PD-NA, Parkinson’s disease without anxiety; HC, healthy controls; LEDD, levodopa equivalent daily dose; H&Y, Hoehn & Yahr scales; UPDRS-III, Unified Parkinson’s Disease Rating Scale; FOGQ, Freezing of Gait Questionnaire; HAMA, Hamilton Anxiety Rating Scale; HAMD, 17-item Hamilton Depression Rating Scale; MMSE, Mini-Mental State Examination; FAB, Frontal Assessment Battery; TIV, total intracranial volume; NA, not applicable. ^a^chi-square test. ^b^one-way analysis of variance (ANOVA). ^c^kruskal-Wallis test. ^d^two-sample *t* test. *represents a significant difference between the PD-A and both the PD-NA and HC groups (*p* < 0.05, corrected by Bonferroni) but not between the PD-NA and HC groups.

### 3.3. Voxel-based morphometry analysis

The VBM analysis showed no statistical gray matter volumetric differences among the PD-A, PD-NA, and HC groups at a significance level of voxel-wise *p* < 0.001 and cluster-wise FWE-corrected *p* < 0.05.

### 3.4. Voxel-based QSM analysis

**Figure 2:**
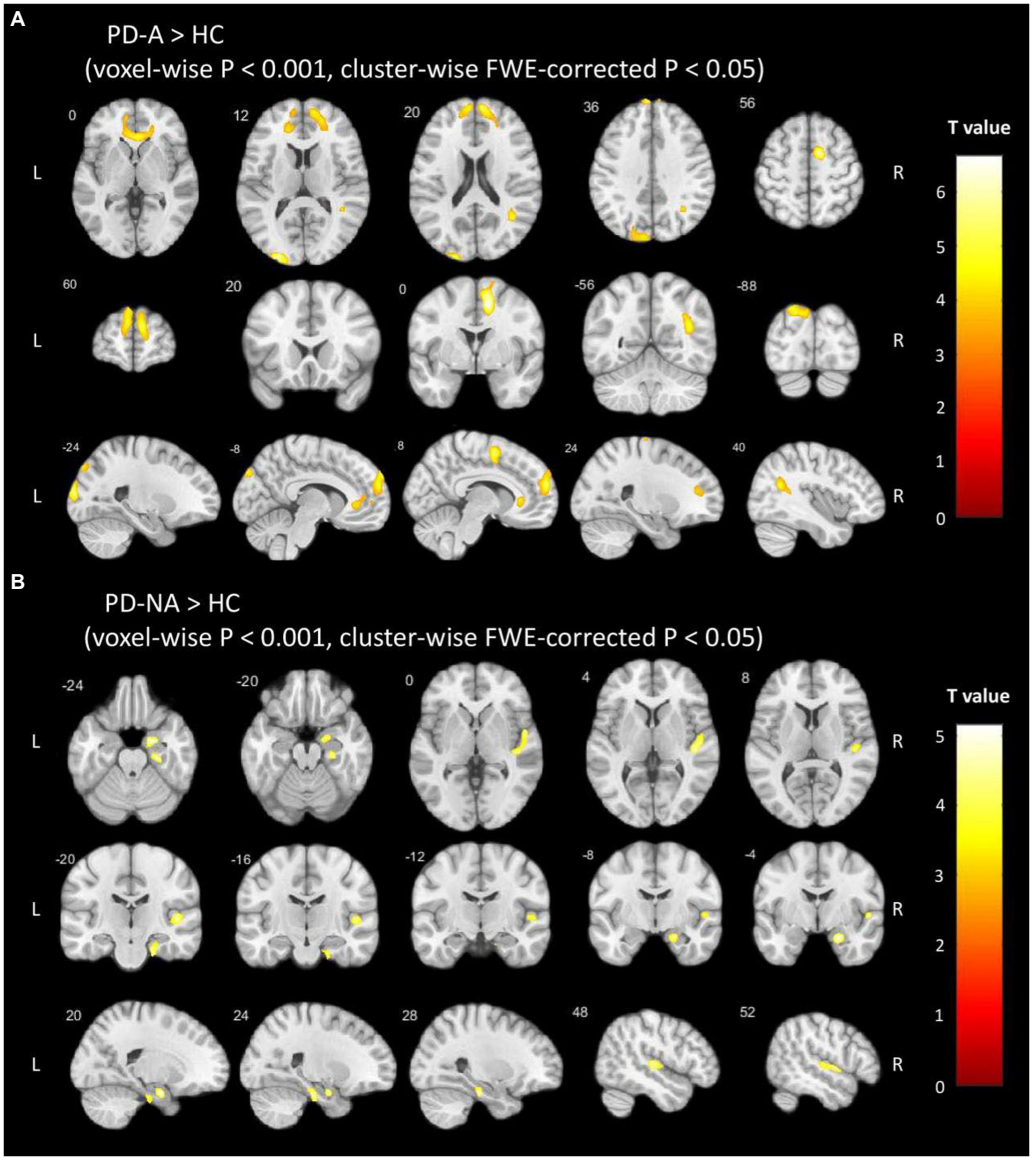
Voxel-based QSM analysis. The clusters with warm color represent significantly **(A)** increased QSM values in the PD-A group than in the HC group and **(B)** increased QSM values in the PD-NA group than in the HC group. FWE, family-wise error; PD-A, Parkinson’s disease with anxiety; PD-NA, Parkinson’s disease without anxiety; HC, healthy control.

According to the voxel-based comparisons of the QSM values among the three study groups, significant differences were found in several brain regions between the PD-A and HC or PD-NA and HC groups, but there was no significant difference between the PD-A and PD-NA groups (voxel-wise *p* < 0.001 and cluster-wise FWE-corrected *p* < 0.05), as shown in Figure 2. The brain regions showing significant differences are shown in Table 2. The PD-A group showed increased QSM values compared to the HC group in the ventral mPFC, ventral ACC, precuneus, angular gyrus, middle occipital gyrus, and supplementary motor area (SMA). Additionally, the PD-NA group showed increased QSM values compared to the HC group in the parahippocampal gyrus and superior temporal gyrus. No significant brain region was found in which the PD-A group showed decreased QSM values compared to the other groups.

**Table 2:** Regions with QSM value differences between each two groups (voxel-wise *p* < 0.001 and cluster-wise FWE-corrected *p* < 0.05).

Regions (AAL)	Side	Cluster size	Peak-point MNI coordinate			
			*X*	*Y*	*Z*	*Z* value
*PD-A increased vs HC*						
mPFC/ACC	R	9,874	8	60	20	5.404
mPFC	L	2,755	−7	59	23	4.907
Precuneus	L	2,101	−20	−88	41	4.755
Angular	R	1787	38	−56	23	5.056
SMA	R	3,757	12	0	49	6.653
*PD-NA increased vs HC*						
Parahippocampal	R	1,605	20	−5	−23	5.152
Superior temporal gyrus	R	1,632	56	−23	3	4.832

QSM, Quantitative Susceptibility Mapping; FWE, family-wise error; AAL, Anatomical Automatic Labeling; MNI, Montreal Neurological Institute; PD-A, parkinson’s disease with anxiety; PD-NA, parkinson’s disease without anxiety; HC, healthy controls; mPFC, medial prefrontal cortex; ACC, anterior cingulate cortex; SMA, supplementary motor area; L, left hemisphere; R, right hemisphere.

### 3.5. ROI-based QSM analysis

**Figure 3:**
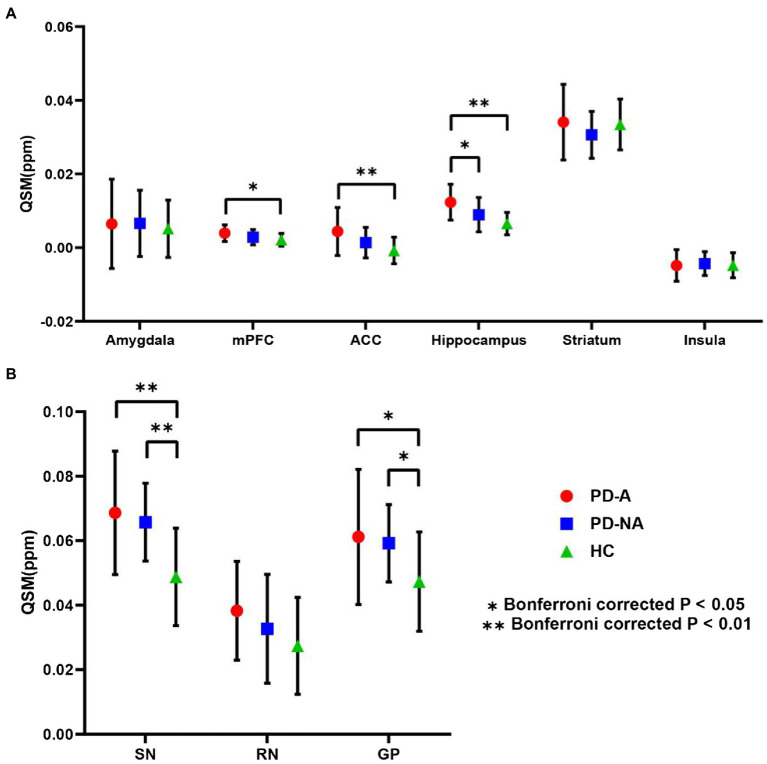
ROI-based QSM analysis. Regional mean QSM values in **(A)** the fear circuit ROIs and **(B)** iron deposition-based ROIs. Error bars signify the standard error of the mean. Stars denote significant differences between the groups: *Bonferroni-corrected *p* < 0.05; **Bonferronicorrected *p* < 0.01. QSM, quantitative susceptibility mapping; ROI, region of interest; PD-A, Parkinson’s disease with anxiety; PD-NA, Parkinson’s disease without anxiety; HC, healthy control; mPFC, medial prefrontal cortex; ACC, anterior cingulate cortex; SN, substantia nigra; RN, red nucleus; GP, globus pallidus.

The mean QSM values of the three study groups in the nine defined ROIs obtained from the AAL template are summarized in Figure 3. Compared to the PD-NA and HC groups, the QSM values of the PD-A group were significantly higher in the hippocampus (*p* = 0.044; *p* < 0.01, Bonferroni correction). In addition, the QSM values of the PD-A group were significantly higher in the ACC (*p* < 0.01) and mPFC (*p* = 0.019) than those in the HC group. Moreover, the QSM values of the PD-A and PD-NA groups were significantly greater than those of the HC group in the SN (*p* < 0.01; p < 0.01) and GP (*p* = 0.024; *p* = 0.034).

The mean QSM values of the three study groups in the six ROIs related to the fear circuit obtained from the Brainnetome Atlas are summarized in Supplementary Figure 1. The results of the six ROIs obtained from the Brainnetome Atlas were similar to those obtained from the AAL template. In detail, the QSM values of the PD-A group were significantly higher in the hippocampus (*p* = 0.048; *p* < 0.01) than those in the PD-NA and HC groups. The QSM values of the PD-A group were significantly higher in the ACC (*p* < 0.01) and mPFC (*p* = 0.010) than those in the HC group. No significant difference was found in the amygdala, striatum, and insula.

### 3.6. Correlation analysis

**Figure 4:**
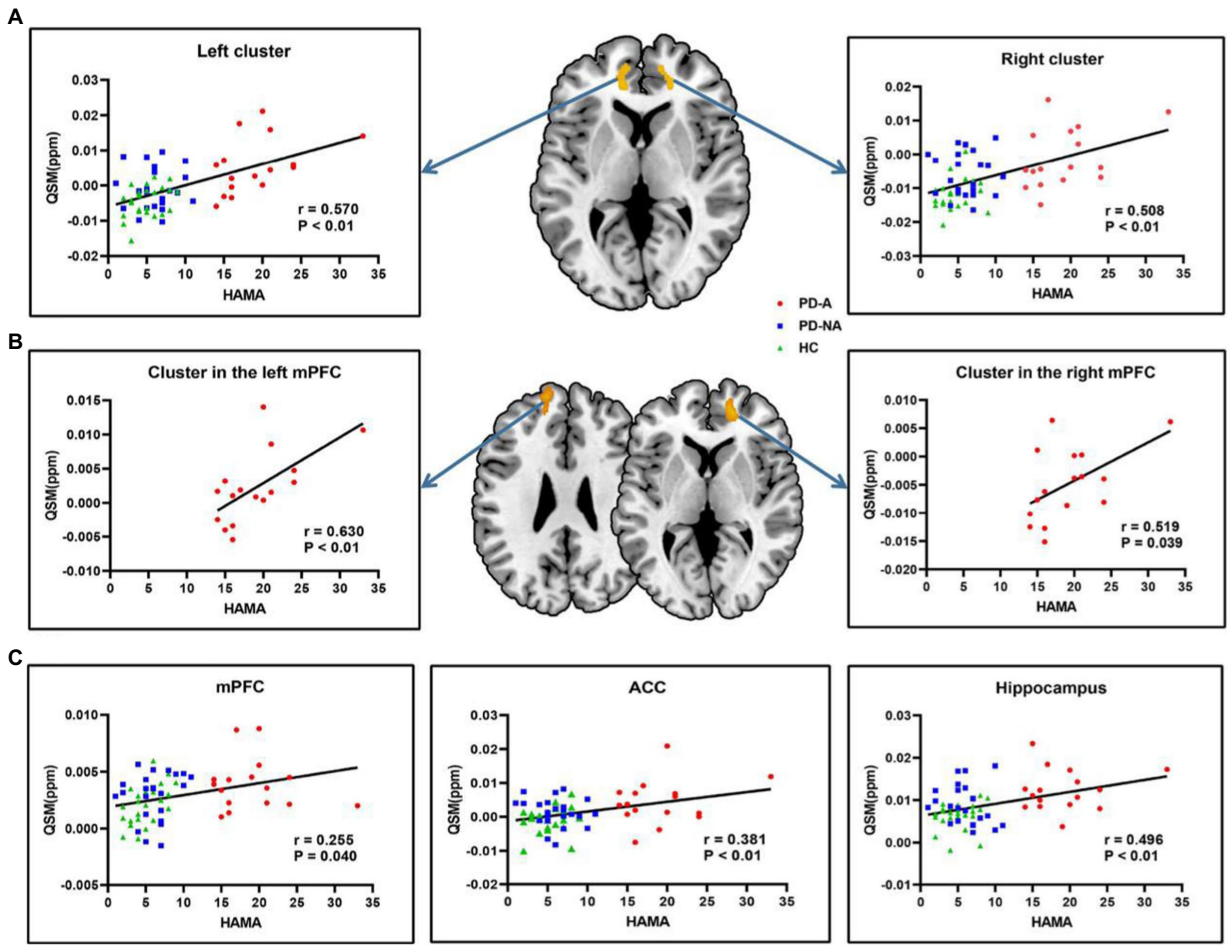
Correlation analysis in all subjects and in the single PD-A group. **(A)** The clusters with warm color represent significantly correlations between voxel-wise QSM values and HAMA scores in all subjects. **(B)** The clusters with warm color represent significantly correlations between voxel-wise QSM values and HAMA scores in the single PD-A group. **(C)** Significantly correlations between regional QSM values and HAMA scores in all subjects. QSM, Quantitative Susceptibility Mapping, HAMA, the Hamilton Anxiety Rating Scale, mPFC, medial prefrontal cortex; ACC, anterior cingulate cortex.

The results of the correlation analysis between voxel-wise QSM values and HAMA scores in all subjects are shown in Figure 4A. Specifically, the two clusters showing significant correlations with HAMA scores were the bilateral ventral mPFC extending to the ACC symmetrically in all subjects (voxel-wise *p* < 0.001 and cluster-wise FWE-corrected *p* < 0.05). According to Pearson’s correlation analysis, significant positive correlations were found between the HAMA scores and the mean QSM values of the left (*r* = 0.570, *p* < 0.01, Bonferroni correction) and right clusters (*r* = 0.508, *p* < 0.01) in all subjects. The results of the correlation analysis between voxel-wise QSM values and HAMA scores in the single PD-A group are shown in Figure 4B. Similar to the results of the correlation analysis in all subjects, the two clusters, respectively, located on the bilateral ventral mPFC showed a significant correlation with HAMA scores in the single PD-A group (voxel-wise *p* < 0.001 and cluster-wise FWE-corrected *p* < 0.05). According to Pearson’s correlation analysis, significant positive correlations were found between the HAMA scores and the mean QSM values of the cluster located on the left mPFC (*r* = 0.630, *p* < 0.01) and the cluster located on the right mPFC (*r* = 0.519, *p* = 0.039) in the single PD-A group.

The results of the correlation analysis between the mean QSM values of defined ROIs and HAMA scores in all subjects are displayed in Figure 4C. Significant positive correlations were found between HAMA scores and the mean QSM values of the mPFC (*r* = 0.255, *p* = 0.040), ACC (*r* = 0.381, *p* < 0.01), and hippocampus (*r* = 0.496, *p* < 0.01) in all subjects. No significant correlations with HAMA scores were found in the amygdala (*r* = 0.027, *p* = 0.833), striatum (*r* = 0.050, *p* = 0.695), insula (*r* = −0.141, *p* = 0.263), SN (*r* = 0.044, *p* = 0.731), GP (*r* = 0.202, *p* = 0.107), and RN (*r* = 0.237, *p* = 0.057). Additionally, no significant correlations were found between the mean QSM values of defined ROIs and HAMA scores in the single PD-A group.

## 4. Discussion

In this study, whole-brain voxel-based QSM analysis and defined ROI-based QSM analysis were combined to identify brain tissue susceptibility alterations related to anxiety in patients with PD. We are the first to report increased brain iron deposition in several areas of the fear circuit in PD patients with anxiety. Specifically, significantly greater mean QSM values in the hippocampus were found in the PD-A group than in the PD-NA group. In addition, compared to the HC group, the PD-A group showed higher QSM values mainly in the fear circuit (mPFC and ACC), SMA, precuneus, angular gyrus, and middle occipital gyrus, while the PD-NA group mainly in the parahippocampal gyrus and superior temporal gyrus. Furthermore, the mean QSM values of the hippocampus, mPFC, and ACC are associated with HAMA scores in the subjects.

In the present study, increased brain iron was observed in the ventral mPFC and ACC in the PD-A group, which was positively correlated with HAMA scores. The ventral mPFC and ACC participate in inhibiting fear conditioning through extinction (Etkin et al., 2011), presumably by promoting adaptation to fearful stimuli to downregulate amygdala activity (Quirk and Mueller, 2008; Mobbs et al., 2009). Moreover, activation of the ventral ACC may be associated with automatic attention disengagement from threats (Vuilleumier et al., 2001). In previous resting fMRI studies, the abnormal amplitude of low-frequency fluctuation (ALFF) activity and functional connectivity (FC) with the amygdala were detected in the ventral mPFC and ACC in PD patients with anxiety (Criaud et al., 2021), and top-down control from the mPFC to the amygdala may be weakened due to dysfunction in these regions in anxiety patients (Kim et al., 2011). In the meantime, as the output nucleus eventually produces anxiety in the fear circuit, abnormal activation of the amygdala was observed in PD patients with anxiety (Dan et al., 2017; Criaud et al., 2021), which may be related to the decreased downregulation of the mPFC and ACC in the amygdala. These neurological dysfunctions may be closely related to dopaminergic system abnormalities in PD. Anxiety symptoms are highly sensitive to dopamine depletion in PD patients (Prediger et al., 2012), which has also been demonstrated in animal studies (Eskow Jaunarajs et al., 2010). Therefore, we considered excessive iron accumulation in the mPFC and ACC may be one of the root causes of anxiety in PD patients.

In addition, we found that excessive iron accumulation also existed in the hippocampus in the PD-A group compared with the other groups, according to the ROI-based QSM analysis. The hippocampus plays an important role in the regulation of emotion during fear conditioning. Along with the amygdala and mPFC, the hippocampus mediates fear conditioning and extinction and is also used to regulate stress responses (Anacker and Hen, 2017). The hippocampus is responsible for encoding and retrieving contextual information or cues related to threats (Bannerman et al., 2014). Decreased control of the hippocampus on contextual retrieval, leading to the recovery of previously vanished fear conditioning, is thought to be one of the mechanisms by which anxiety disorders develop (Rauch et al., 2006). In addition, the hippocampus is involved in assessing the threat level of stimulation (Mah et al., 2016). For example, a recent animal study showed that the hippocampus is involved in the response to acute stress (von Ziegler et al., 2022), suggesting that changes in the hippocampus may lead to an abnormal threat response. Several anatomical MRI studies have found that hippocampal volume is negatively correlated with anxiety scores in patients with anxiety (Irle et al., 2010). In contrast, no morphological changes in the hippocampus were found in the PD-A group, suggesting that iron deposition in the hippocampus of the PD-A group had increased even before morphological changes occurred, supporting the idea that QSM was more sensitive to earlier alterations of brain tissue than conventional anatomical MRI measurements in PD patients with anxiety. Thus, excessive iron accumulation in the hippocampus may be closely linked to anxiety in PD.

Anxiety may be caused by an imbalance in the fear circuit (Mah et al., 2016). In other words, whether the ventral neural system (amygdala) is highly active or the dorsal nervous system (mPFC, ACC, and hippocampus) control of the amygdala is weakened, this may cause anxiety. Our findings seem to support the hypothesis that damage to the dorsal nervous system may be caused by excessive iron accumulation. Pathologically, iron tends to accumulate in Lewy bodies (Castellani et al., 2000), which is a gradual neurodegenerative process that leads to the redistribution of iron in the brain in PD. Excessive iron accumulation has been suggested to cause oxidative stress in brain cells, resulting in DNA damage, mitochondrial damage, and lipid oxidative damage in dopaminergic neurons, ultimately leading to their death (Ostrerova-Golts et al., 2000; Sian-Hülsmann et al., 2011). Then, excessive iron accumulation in the dorsal nervous system disrupts the downregulation of the ventral nervous system rather than spontaneous overactivation of the ventral nervous system, which causes anxiety in PD patients. Therefore, we thought that the root cause of the high lifetime risk of anxiety in PD patients may be the redistribution of brain iron due to neurodegeneration.

Incidentally, widespread increased susceptibility alterations in the default mode network (DMN), including the precuneus, angular, mPFC, and ACC, were also found in this study. Decreased functional connectivity within the DMN has been reported in PD patients with anxiety (De Micco et al., 2021), but this contrasts with another study on social anxiety (Arnold Anteraper et al., 2014). The damage caused by the abnormal susceptibility alterations within the DMN may explain the difficulty of anxiety patients in focusing attention on the self and away from goal-oriented stimuli (De Micco et al., 2021). In addition, although striatal abnormalities have been discovered in many previous neuroimaging studies of anxiety in PD, whether morphological (Oosterwijk et al., 2018), functional (Wang et al., 2017), or metabolic (Ceravolo et al., 2013; Bayram et al., 2020), no related susceptibility changes were observed in this study. These striatal abnormalities may be related to secondary dopamine depletion resulting from damage to the mPFC and ACC.

The increased QSM value of the SMA may be associated with postural instability in PD (Pinson et al., 2022). The association between anxiety and motor symptoms in PD remains controversial (Prediger et al., 2012). Freezing of gait seems to be more strongly associated with anxiety in PD (Ehgoetz Martens et al., 2014; Gilat et al., 2018). However, in other studies of PD patients with anxiety, no similar relationship between anxiety and motor symptoms was found (Arnold Anteraper et al., 2014; Carey et al., 2020). Statistically, our results support the idea that anxiety has no influence on motor symptoms. We speculated that this difference may stem from the timing of anxiety symptoms in PD: anxiety as a precursor symptom before PD and anxiety that progresses continuously after PD may have different neural mechanisms (Dissanayaka et al., 2014).

It should be emphasized that, in this study, significantly increased iron deposition in the hippocampus was shown using the ROI-based QSM analysis, but no corresponding changes were found in the whole-brain voxel-based QSM analysis. We attributed this difference to the technical limitations of the voxel-based analysis: on one hand, the voxel-based analysis may lack the ability to reliably assess too small brain regions such as the SN and hippocampus; on the other hand, possible errors in spatial normalization may lead to some extent of spatial uncertainty in the results, although spatial smoothing had been applied to minimize this spatial uncertainty (Uchida et al., 2019). Therefore, while voxel-based QSM analysis is a comprehensive and convenient method of analysis, ROI-based QSM analysis can more sensitively and accurately assess susceptibility alterations in brain ROIs. Although we detected significant susceptibility differences in the mPFC and ACC between the PD-A and HC groups, no difference was observed between the PD-A and PD-NA groups. This may be because several subjects in the PD-NA group had HAMA scores close to the critical value of 12. In other words, the subjects in the PD-NA group could not be classified as the PD-A group but could possibly progress to anxiety according to the grouping criteria in this study, which may lead to reduced statistical differences. This conformed to the theory that the lifetime risk of anxiety is approximately 60% (Martínez-Martín and Damián, 2010) and is a possible explanation for the longer duration of PD in the PD-A group than in the PD-NA group in this study, which was consistent with previous research (Dissanayaka et al., 2014). Thus, we believe that the role of increased brain iron in the mPFC and ACC in the neural mechanisms of anxiety in PD should be valued.

This study has some limitations in addition to the ones listed above. First, limited by the comparatively small sample size, this study was only a preliminary exploration of the possible neural mechanisms of anxiety in PD, while a comparison between subtypes of anxiety in PD was impossible. Second, regarding previous studies (Wang et al., 2017), only the HAMA scale was used in the assessment of anxiety in this study, which was too simple to comprehensively assess anxiety severity. Future studies should use a combination of multiple anxiety scales. Third, although screenings were conducted and patients with obvious depression were excluded from the PD clinic, the HAMD score of PD-A subjects was statistically superior to that of other subjects, which was consistent with the high comorbidity of anxiety and depression in PD (Martínez-Martín and Damián, 2010). This may be related to some overlap in scoring rules between the HAMD and HAMA scales. Despite taking the HAMD score as a covariate, its effects on the results could not be completely ruled out. Fourth, the serum iron and transferrin levels of the subjects were not collected, which makes it impossible to study the relationship between serum iron and transferrin levels with iron deposition in the brain. In the future, we would add the collection of these clinical indicators in the enrollment of subjects. Fifth, due to the limitation of 3.0 T MRI in studying small structures in the brain (Wang et al., 2019), the application of 7.0 T MRI in this study may provide more information in the future. Finally, this was a cross-sectional study in which the follow-up of the subjects was not completed. Therefore, the longitudinal development of anxiety symptoms in patients with PD, especially PD-NA subjects close to the anxiety grouping criteria, could not be investigated at present. We would make a great effort to further study the neural mechanism of the occurrence and development of anxiety in PD after completing follow-up work.

## 5. Conclusion

Based on a review of the literature, this study, most likely for the first time, revealed the distribution pattern of brain iron and excessive iron accumulation in the fear circuit of PD patients with anxiety, providing a new approach to explaining the neural mechanism of anxiety in PD. However, the hypothesis proposed in this study may only be used to explain progressive anxiety after PD, and further studies are needed to explain the similarities and differences between the neural mechanisms of precursor anxiety and progressive anxiety in PD.

## Data availability statement

The raw data supporting the conclusions of this article will be made available by the authors, without undue reservation.

## Ethics statement

The studies involving human participants were reviewed and approved by the Ethics Committee of Wuxi People’s Hospital. The patients/participants provided their written informed consent to participate in this study.

## Author contributions

XF, FW, XH, KaC, and LZ: study concept and design. KaC, LZ, HM, KeC, and YS: data collection. KaC: data analysis and manuscript writing. XF, XH, LZ, and XM: project development and manuscript revision. All authors contributed to the article and approved the submitted version.

## Funding

This study was supported by the Jiangsu Province Natural Science Foundation (No. BK20191143, XF), the National Natural Science Foundation of China (No. 81271629, XF), the Medical Expert Team Program of Wuxi Taihu Talent Plan 2021, and the Top Talent Support Program for young and middle-aged people of Wuxi Health Committee (No. BJ2020015, XH).

## Conflict of interest

The authors declare that the research was conducted in the absence of any commercial or financial relationships that could be construed as potential conflicts of interest.The reviewer YY declared a shared parent affiliation with the authors to the handling editor at the time of review.

## Publisher’s note

All claims expressed in this article are solely those of the authors and do not necessarily represent those of their affiliated organizations, or those of the publisher, the editors and the reviewers. Any product that may be evaluated in this article, or claim that may be made by its manufacturer, is not guaranteed or endorsed by the publisher.

## Figures and Tables

**Table:** 

3D	Three-dimensional
AAL	Anatomical Automatic Labeling
ACC	Anterior cingulate cortex
ALFF	Abnormal amplitude of low-frequency fluctuation
ANCOVA	Analysis of covariance
ANOVA	Analysis of variance
CAT	Computational Anatomy Toolbox
DMN	Default mode network
DWI	Diffusion-weighted
FAB	Frontal Assessment Battery
FLAIR	Fluid-attenuated inversion recovery
fMRI	Functional magnetic resonance imaging
FOGQ	Freezing of Gait Questionnaire
FWE	Family wise error
GP	Globus pallidus
H&Y	Hoehn & Yahr
HAMA	Hamilton Anxiety Rating Scale
HAMD	Hamilton Depression Rating Scale
HC	Healthy control
LEDD	Levodopa equivalent daily dose
MDS-UPDRS-III	Movement Disorder Society Unified PD Rating Scale motor part 3
MMSE	Mini-Mental State Examination
MNI	Montreal Neurological Institute
mPFC	Medial prefrontal cortex
MP-RAGE	Magnetization prepared rapid acquisition gradient echo
MRA	Magnetic resonance angiography
MRI	Magnetic resonance imaging
PD	Parkinson’s disease
QSM	Quantitative susceptibility mapping
RN	Red nucleus
SPSS	Statistical Product Service Solutions
ROI	Region of interest
SMA	Supplementary motor area
SN	Substantia nigra
STAR	Streaking artifact reduction
TIV	Total intracranial volume
VBM	Voxel-based morphometry

## Footnotes

^1^ https://people.eecs.berkeley.edu/~chunlei.liu/software.html

^2^www.fmrib.ox.ac.uk/fsl, v 6.0.1

^3^ https://github.com/ANTsX/ANTs/wiki

^4^ http://atlas.brainnetome.org
