# Supplementary material for: The impact of iron deposition on the fear circuit of the brain in patients with Parkinson’s disease and anxiety
Source: Front Aging Neurosci. 2023 Feb 9;15:1116516. doi: 10.3389/fnagi.2023.1116516 (PMC9951615; doi:10.3389/fnagi.2023.1116516)
Supplement: Supplementary file 2 [file Table_1.DOCX]

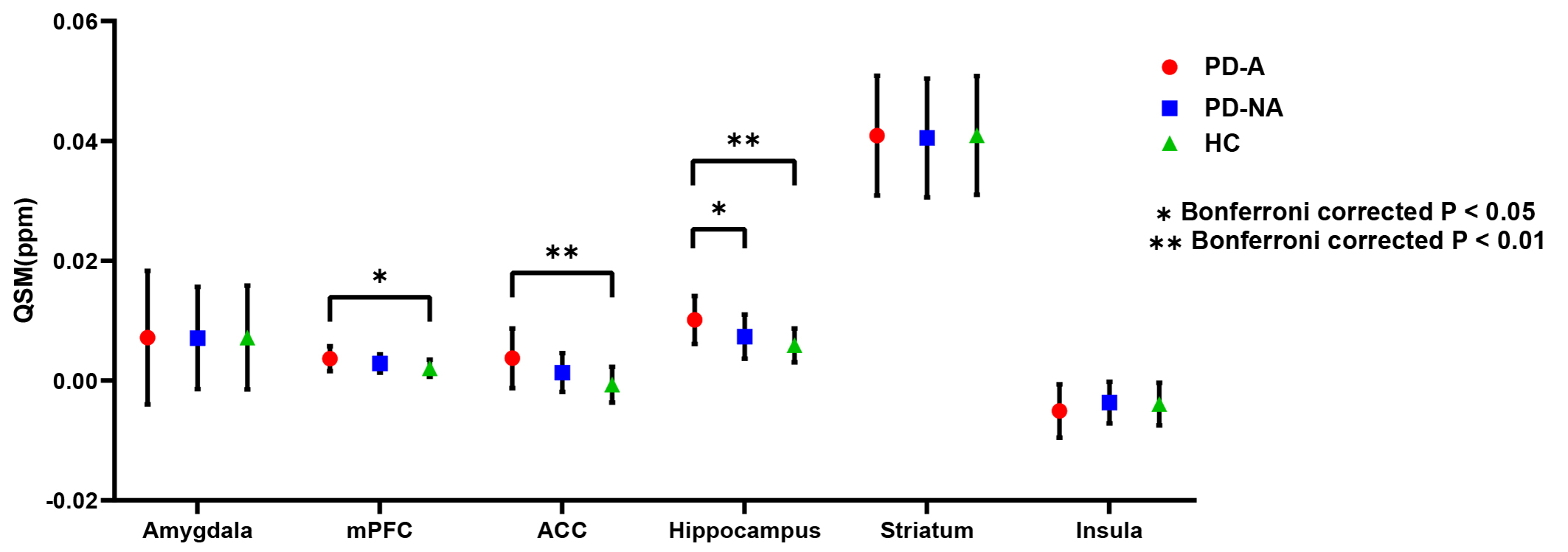


**Supplementary Figure 1.** ROI-based QSM analysis. Regional mean QSM values in the fear circuit ROIs obtained from the Brainnetome Atlas. Error bars signify the standard error of the mean. Stars denote significant differences between the groups: *Bonferroni-corrected P < 0.05; **Bonferronicorrected P < 0.01. QSM, quantitative susceptibility mapping; ROI, region of interest; PD-A, Parkinson’s disease with anxiety; PD-NA, Parkinson’s disease without anxiety; HC, healthy control; mPFC, medial prefrontal cortex; ACC, anterior cingulate cortex.
